# Green, Cost-Effective Simultaneous Assay of Chloramphenicol, Methylparaben, and Propylparaben in Eye-Drops by Capillary Zone Electrophoresis

**DOI:** 10.1155/2021/5575701

**Published:** 2021-04-10

**Authors:** Thi Thanh Vuong Tong, Thi Thoa Cao, Nguyen Ha Tran, Thi Kim Van Le, Dinh Chi Le

**Affiliations:** ^1^Department of Analytical Chemistry and Toxicology, Hanoi University of Pharmacy, Hanoi, Vietnam; ^2^National Institute of Medicinal Materials, Hanoi, Vietnam; ^3^National Institute of Pharmaceutical Technology, Hanoi University of Pharmacy, Hanoi, Vietnam

## Abstract

A green, cost-effective, and simple capillary zone electrophoresis (CZE) method was developed and validated for simultaneous determination of chloramphenicol, methylparaben, and propylparaben in eye-drops. With sodium tetraborate as background electrolyte (BGE), the apparent mobilities of chloramphenicol, methylparaben, and propylparaben increased and analysis time reduced when pH of BGE increased from 8.5 to 10.0 and concentration of BGE decreased from 40 mM to 15 mM, but complete separation of chloramphenicol from other matrix components was achieved only with sodium tetraborate concentration at 30 mM or higher and at pH = 9.3 or lower. The most suitable electrophoretic conditions for the intended application were a 30 mM sodium tetraborate solution, pH 9.3 as BGE, working voltage set at 25 kV, and UV detection at 280 nm at the cathodic extremity of the capillary. The final method was validated and proved to be reliable for assay of chloramphenicol, methylparaben, and propylparaben in eye-drops.

## 1. Introduction

Chloramphenicol is a broad-spectrum antibiotic effective against both Gram-positive and Gram-negative bacteria [1]. Due to its potential grave side effect (bone marrow aplasia) [1, 2], chloramphenicol is used mainly for local treatment of eye infections in form of eye-drops or ointments. Parabens are esters of p-hydroxybenzoic acid commonly used as antimicrobial preservatives thanks to their antibacterial activities [3]. However, in pharmaceutical preparations, only methylparaben and propylparaben are currently used as preservatives due to concerns regarding their safety [4]. So far, UV-Vis spectroscopy has been used for assay of chloramphenicol in pharmaceutical-grade raw material [5]; the high-performance liquid chromatography (HPLC) method using C18 column has been developed for the assay of chloramphenicol in pharmaceutical dosage forms [5] and biological sample [6]; and capillary zone electrophoresis (CZE) with amperometric detection has been used for quantifying chloramphenicol in blood [7] and eye-drops [8]. More recently, chloramphenicol in eye-drops was determined by the electrochemical method using a glassy carbon electrode modified by Ho^3+^/Co_3_O_4_ nanoflowers [9]. Propylparaben and methylparaben have been analyzed simultaneously in cosmetic products with reversed-phase HPLC [10], in pharmaceutical and cosmetic products with capillary electrophoresis with conventional UV detection [11], and in pharmaceutical preparations by microchip electrophoresis with conductivity detection [12]. Chloramphenicol and methylparaben were determined by UV spectrometry with multivariate calibration [13]. So far, HPLC is the most common technical choice for assay of chloramphenicol, propylparaben, and methylparaben, though no method for simultaneous analysis of these substances has been published up to now within the limit of our bibliography research. Despite many advantages provided by HPLC, the operational cost of this technique is quite high, particularly in large-scale analysis, such as in routine quality control or postmarketing surveillance of pharmaceutical products, for consuming high-quality solvents and chemicals and the cost for analytical columns. Most of the HPLC methods use organic solvents in mobile phases, which end up as organic waste, which is often a pollutant for the environment and hazardous for human health. In comparison to HPLC, CZE uses mostly aqueous electrolyte solutions in small quantities and low-cost fused-silica capillaries, which are much cheaper than high-quality organic solvents and chromatography columns, leading to much lower operational cost, material consumption, and, last but not less, no hazardous waste after analysis. Therefore, this technique is very cost-effective and friendly with the environment, suitable for routine analysis of relatively simple samples containing ionized or ionizable analytes. Employing more extensively CZE-based methods can help quality control laboratories with limited resources in developing countries like Vietnam work more effectively in reserving the more robust and capable HPLC techniques for the most challenging analytical problems. In this study, a simple, cost-effective, and green capillary zone electrophoresis (CZE) method was developed for simultaneous assay of chloramphenicol, methylparaben, and propylparaben in eye-drops. The method was fully validated according to the current demands of ICH [14] and AOAC International [15], aiming at providing a reliable tool for use in routine quality control of eye-drops containing these analytes.

## 2. Materials and Methods

### 2.1. Instrumentation

3D-CE capillary electrophoresis system of Agilent Technologies (Santa Clara, CA, USA) was used for method development and validation. This system was equipped with a PDA detector. The capillary zone electrophoresis separation was executed on a fused-silica capillary (total length: 35 cm, effective length: 30 cm, and inner diameter: 50 *µ*m). Software ChemStation version B.03.01 of Agilent Technologies (Santa Clara, CA, USA) was used for data processing.

To measure and adjust the pH of sodium tetraborate solution, a pH meter CyberScan pH 510 of Eutech Instruments was used (Eutech Instruments Pte. Ltd., Singapore).

### 2.2. Chemicals and Reagents

Reference standards of chloramphenicol (purity 99.6%), methylparaben (purity 100.0%), and propylparaben (purity 99.9%) were purchased from the National Institute of Drug Quality Control (Hanoi, Vietnam). Chloramphenicol eye-drops (containing nominally 32 mg of chloramphenicol, 88 mg of boric acid, 16 mg of sodium borate, 16 mg of sodium chloride, 1.44 mg of methylparaben, and 0.16 mg of propylparaben per vial of 8 ml) was purchased from the market. Sodium tetraborate, sodium hydroxide, and hydrochloric acid PA grade were purchased from Merck Vietnam (Ho Chi Minh City, Vietnam). All solutions were prepared by using deionized water. In case of sodium tetraborate solution, the pH was adjusted, whenever necessary, with 0.1 M hydrochloric acid solution or 0.1 M sodium hydroxide solution.

### 2.3. Electrophoretic Conditions

The background electrolyte was a 30 mM solution of sodium tetraborate, pH 9.3. The analysis was carried out on an Agilent 3D-CE capillary electrophoresis apparatus equipped with a PDA detector set at 280 nm for recording electropherograms. The electrophoretic separation was conducted on a fused-silica capillary (total length: 35 cm, effective length: 30 cm, and inner diameter: 50 *µ*m) maintained at 25°C with a voltage of 25 kV applied on the two extremities of the capillary. The detection was done at the cathodic extremity of the capillary. The injection was done hydrodynamically on the anodic extremity of the capillary by applying a pressure of 50 mbar in 15 seconds. Before each analysis, the capillary was prewashed consecutively in 2 minutes with deionized water, 1 minute with 0.1 M solution of sodium hydroxide, 2 minutes with deionized water, and 2 minutes with background electrolyte. Detailed information about the selection of electrophoretic and prewash conditions was provided in section 3.1.

### 2.4. Preparation of Standard Solution

Stock standard solutions of chloramphenicol (about 4000 *µ*g/ml), methylparaben (about 1000 *µ*g/ml), and propylparaben (about 1000 *µ*g/ml) were prepared by dissolving an accurately weighed quantity of corresponding reference standard in deionized water. Mix standard solutions for routine analysis and method validation were prepared by accurate dilution of stock standard solutions to the intended concentration with deionized water. Working concentrations in mix standard solution for routine analysis were about 2000 *µ*g/ml for chloramphenicol, 90 *µ*g/ml for methylparaben, and 10 *µ*g/ml for propylparaben. For method validation, 5 mix standard solutions having concentrations of each analyte at about 60.0%, 80.0%, 100.0%, 120.0%, and 140.0% of respective working concentration were prepared for linearity study. Standard solutions were filtered through 0.2 *µ*m membrane filter before used for electrophoretic analysis.

### 2.5. Preparation of Sample Solution

To prepare the sample solution for routine analysis, one volume of eye-drops was diluted with one volume of deionized water. For method specificity evaluation, a solution in deionized water containing 88 mg of boric acid, 16 mg of sodium borate, and 16 mg of sodium chloride per 8 ml was used as a placebo. For the recovery study, different portions of placebo were spiked with chloramphenicol, methylparaben, and propylparaben at 80.0%, 100.0%, and 120.0% of working concentration for each analyte (see 2.4). Three spiked solutions were prepared independently for each spiked level. Sample solutions were filtered through a 0.2 *µ*m membrane filter before used for electrophoretic analysis.

### 2.6. Method Validation

#### 2.6.1. Specificity

The reliability of an analytical method depends firstly on its specificity, that is, the ability to distinguish between the analyte(s) and the other components in the sample matrix [16]. In this CZE method, it is assured by complete separation of peaks corresponding to chloramphenicol, methylparaben, and propylparaben from each other and from other possible peaks originating from the sample matrix. Specificity evaluation was done by injecting solutions of standard, sample, and placebo into the electrophoretic system.

#### 2.6.2. Linearity

To evaluate the linearity of the method, mixed standard solutions prepared as described in 2.4 were injected into the capillary and analyzed. The linearity between concentration and peak area of each analyte was evaluated using the least square linear regression method, and the significance of linear regression was confirmed by a one-way ANOVA test if *P* < 0.05.

#### 2.6.3. Sensitivity

According to the current guideline Q2R1 of ICH [14], determining the limit of detection (LOD) and limit of quantification (LOQ) is not required for quantitative methods. However, it is necessary to assure that the LOQ of the method for each analyte was below the working concentration. Therefore, the limit of quantitation (LOQ) of each analyte was determined by analyzing solutions having different concentrations of chloramphenicol, methylparaben, and propylparaben and measuring the signal-to-noise ratio for each analyte. The limit of quantitation (LOQ) is the concentration giving a signal-to-noise ratio of about 10 : 1 with RSD of less than 10% with triplicate analysis [17].

#### 2.6.4. Accuracy

The accuracy of the method was evaluated by the recovery rate of chloramphenicol, methylparaben, and propylparaben from a placebo solution spiked with these analytes (as described in 2.5) [18].

#### 2.6.5. Precision

The precision of the CZE method was validated in terms of system suitability, repeatability, and intermediate precision [14–16, 19].

The system suitability was determined by six measurements of a mixed standard solution containing each analyte at 100% of working concentration on the same day [15]. Repeatability and intermediate precision were determined by six measurements of a sample solution containing each analyte at approximately 100% of working concentration on the same day and on two different days, respectively.

#### 2.6.6. Robustness

The robustness of the method was verified by assessing the variation of results after minor changes in the experimental conditions [20]. In this study, the following changes were made:

- Voltage: ± 2 kV.
- Injection pressure: ± 5 mbar.

At each condition, mixed standard solution of chloramphenicol, methylparaben, and propylparaben and sample solutions at 100% of working concentration were injected into capillary. The robustness of the method was evaluated from the RSD of peak area for each analyte after three consecutive injections of standard solution and the RSD of the content of chloramphenicol, methylparaben, and propylparaben from sample solutions.

## 3. Results and Discussion

### 3.1. Method Development and Optimization

#### 3.1.1. Selection of Background Electrolyte

**Figure 1:**
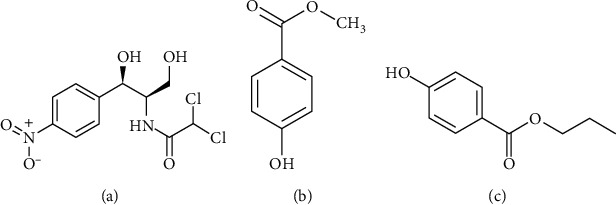
Structural formulas of chloramphenicol (a), methylparaben (b), and propylparaben (c).

In this study, sodium tetraborate was used to prepare the background electrolyte solution. It has a value of pKa of about 9; therefore, it produces an electroosmotic flow (EOF) toward cathode in a fused-silica capillary due to the deprotonation of silanol group on the inner surface [21]. At pH from 6.5 upward, the parabens (Figures 1(b) and 1(c)), began to deprotonate and became negatively charged, and pKa values of both methylparaben and propylparaben were about 8.47 [22]. Chloramphenicol, in contrast, was itself neutral in the pH range up to 11 [23], but tetraborate was known to form negatively charged complex with hydroxyl groups of polyol substances [24], including chloramphenicol (Figure 1(a)) [25] in aqueous solution. Therefore, in sodium tetraborate solution, chloramphenicol, methylparaben, and propylparaben were anions and migrated toward cathode with apparent mobility slower than that of EOF, and they can be simultaneously separated and detected at the cathode side of the capillary. To obtain suitable electrophoretic conditions for the final method, the concentration of sodium tetraborate was varied from 15 mM to 40 mM and the pH was varied from 8.5 to 10.0. The measured pH of unadjusted sodium tetraborate solutions from 15 mM to 40 mM varied within the range of 9.3 ± 0.1, so the influence of pH was investigated at 8.5 (near the pKa of methylparaben and propylparaben), 9.3 (around original pH), and 10.0 (higher than the original pH). The working voltage was investigated from 5 kV to 25 kV, taking into account the maximum acceptable current (not exceeding 100 *µ*A) imposed by the manufacturer of the CE apparatus.

**Figure 2:**
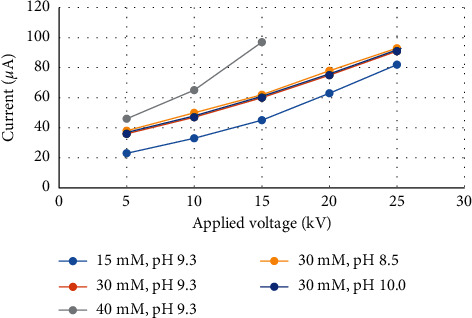
Relationship between the current and applied voltage at different concentrations and pH of sodium tetraborate solution.

**Figure 3:**
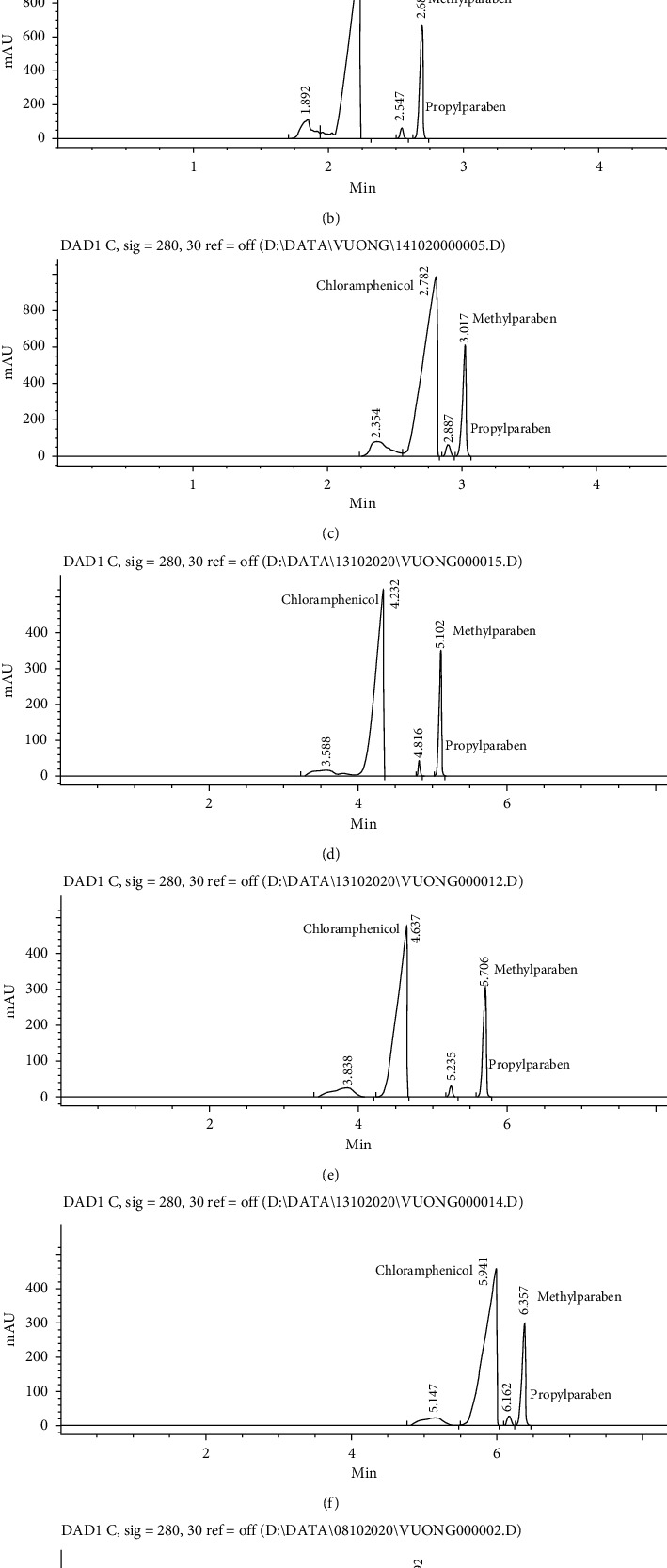
Electropherograms at different concentrations and pHs of sodium tetraborate solution (a): 15 mM, pH = 10; (b): 15 mM, pH = 9.3; (c): 15 mM, pH = 8.5; (d): 30 mM, pH = 10; (e): 30 mM, pH = 9.3; (f): 30 mM, pH = 8.5; and (g): 40 mM, pH = 9.3.

In terms of working voltage, a preliminary study on different concentrations and pH of sodium tetraborate solutions pointed out that, at a concentration of 40 mM, the working voltage cannot be set higher than 15 kV, whereas, at 15 mM and 30 mM, the working voltage can go up to 25 kV, as shown in Figure 2. When the concentration of sodium tetraborate was fixed, pH variation from 8.5 to 10.0 did not affect the range of applicable working voltage (see Figure 2). In consequence, with 40 mM sodium tetraborate solution, lower maximum voltage led to longer analysis time (Figure 3(c)) comparing to analysis times obtained with 15 mM (Figures 3(a)to 3(c)) and 30 mM solutions (Figures 3(d)to 3(f)).

**Figure 4:**
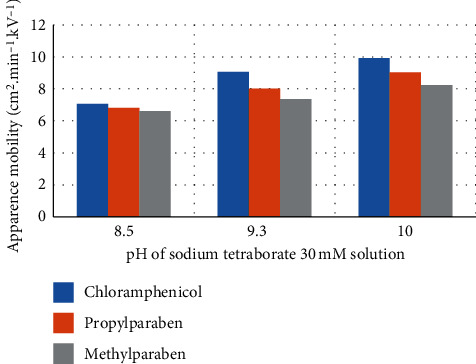
Relationship between the apparent mobility of analytes and pH of sodium tetraborate solution.

**Figure 5:**
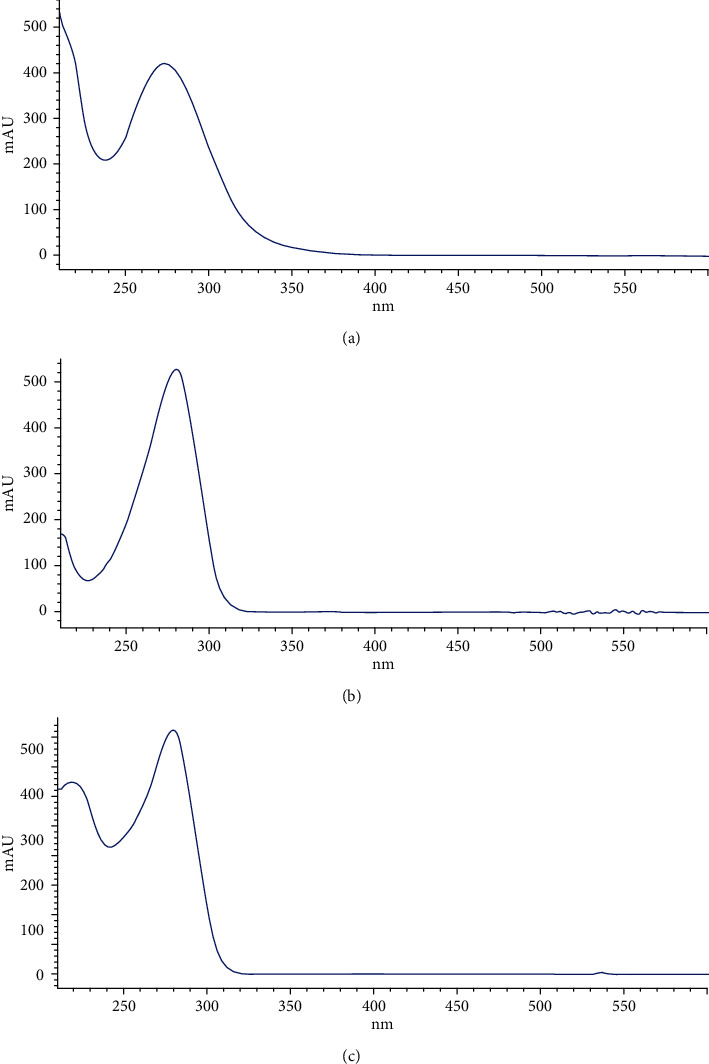
UV spectra of chloramphenicol (a), methylparaben (b), and propylparaben (c).

**Figure 6:**
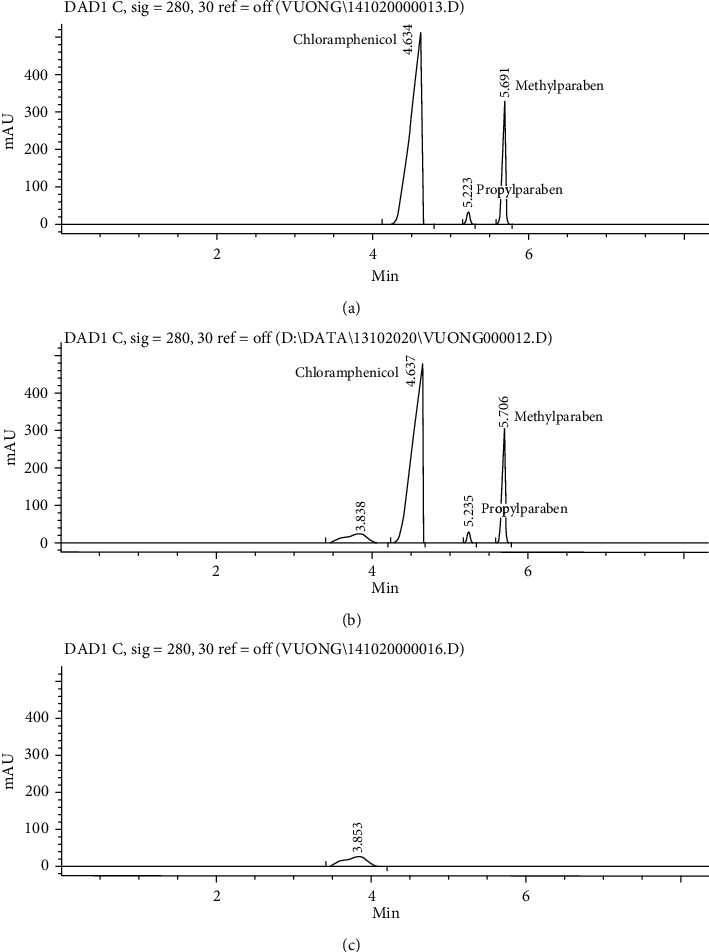
Typical electropherograms of mixed standard solution (a), eye-drops (b), and placebo (c).

In all investigated conditions of BGE, the migration order among the analytes remained the same, chloramphenicol had the shortest migration time, followed by propylparaben, and methylparaben had the longest migration time (as shown in the electropherograms of Figure 3). The apparent mobilities of the three analytes increased when the pH increased from 8.5 to 10.0 at the same concentration of sodium tetraborate (Figure 4) due to the increase of EOF. Therefore, at the same concentration of sodium tetraborate, analysis time was the shortest at pH = 10.0 and the longest at pH = 8.5 (see Figures 3(a)to 3(c) at 15 mM and Figures 3(d)to 3(f) at 30 mM). Similarly, apparent mobilities of all analytes increased when the concentration of sodium tetraborate reduced from 30 mM (Figures 3(d)to 3(f)) to 15 mM (Figures 3(a)to 3(c)) when working voltage was set at 25 kV.

The three analytes were well separated among them, but the resolution reduced drastically at pH 8.5. At this pH, close to the pKa of methylparaben and propylparaben, perhaps because their ionization reduced, their apparent mobilities were closer to one another and closer to that of chloramphenicol than at pH = 9.3 or 10 (Figure 4), causing the loss in resolution.

While the separation of chloramphenicol from propylparaben and methylparaben was easily obtained, the separation between chloramphenicol and other matrix components of the eye-drops which migrated just before the peak of chloramphenicol was more difficult. The peak of chloramphenicol was completely separated with 30 mM sodium tetraborate solution at pH = 9.3 or 8.5 (Figures 3(e) and 3(f)) and with 40 mM sodium tetraborate solution (Figure 3(g)). In contrast, no complete separation was achieved with 15 mM sodium tetraborate solution at pH from 8.5 to 10.0 (see Figures 3(a)to 3(c)). With 30 mM sodium tetraborate at pH 10.0 (Figure 3(d)), stronger EOF and higher apparent mobility of chloramphenicol led to shorter migration time but were insufficient in mobility difference with some matrix component(s), and the peak of chloramphenicol was not completely separated.

So, for these particular eye-drops, due to the electrophoretic characteristics of its components, the most suitable background electrolyte was 30 mM sodium tetraborate solution, pH 9.3, because it provided complete separation of chloramphenicol, propylparaben, and methylparaben from each other and from other matrix components at the shortest time (less than 6 minutes, as shown in Figure 3(e)). This pH was also close to the original pH of 30 mM sodium tetraborate solution; therefore, the pH adjustment of background electrolyte would be easier in routine application.

The experimental results also pointed out that quicker simultaneous assay of chloramphenicol, methylparaben, and propylparaben may be possible with 15 mM sodium tetraborate solution at pH = 10 in about 2.6 minutes (Figure 3(a)) in case of a sample with less matrix interference or in combination with a more elaborate sample preparation procedure (like an extraction step to isolate analytes from sample matrix). But in the latter case, any gain in analysis time might be compromised by the need of more time-consuming sample preparation.

#### 3.1.2. Selection of Detection Wavelength

The UV-Vis spectra of chloramphenicol, methylparaben, and propylparaben measured at the detection window of the capillary are shown in Figure 5. Due to their structural similarity, both methylparaben (Figure 5(b)) and propylparaben (Figure 5(c)) had an absorption maximum at 280 nm, whereas chloramphenicol (Figure 5(a)) had an absorption maximum at 272 nm. Because, in the eye-drops, the concentration of chloramphenicol was much higher than those of methylparaben and propylparaben, the wavelength for recording electropherograms was selected at 280 nm to give priority to analytes with lower concentrations in sample.

#### 3.1.3. Selection of Prewash Program between Analyses

The key point for the success of a capillary electrophoresis method is maintaining the repeatability of migration time and peak response of analytes from one injection to another. The investigation of prewash conditions pointed out that only a 4-step prewash procedure using consecutively deionized water, sodium hydroxide 0.1 M, deionized water, and background electrolyte before each injection gave repeated migration time for all three analytes (with RSD < 2.0%; see also information regarding system suitability in Table 1).

### 3.2. Method Validation

The final CZE method was fully validated according to the current requirements of ICH guideline Q2R1 [13] for the assay method. In terms of specificity, the validation was performed by comparing the electropherograms of placebo solution, standard solution, and sample solution after being injected separately into the CE system, and the results are shown in Figures 6(a)–6(c). In selected electrophoretic conditions, three analytes were completely separated, the peak of chloramphenicol was the first to arrive at the detection window, followed by that of propylparaben, and lastly, that of methylparaben (Figure 6(a)). In the electropherogram of a placebo, no peak appeared at migration times of chloramphenicol, propylparaben, and methylparaben (Figure 6(c)), whereas the electropherogram of eye-drops giving three peaks corresponding to those of chloramphenicol, propylparaben, and methylparaben was obtained with the standard solution in terms of migration times (Figure 6(b)). These results indicate that the electrophoretic conditions employed in this method were specified enough to separate the three analytes from one another as well as from other compositions of the eye-drops.

The quantitative validation results summarized in Table 1 also proved that the method met the current requirements of performance in terms of system suitability, linearity, precision (repeatability, intermediate precision), and accuracy when being applied for simultaneous assay of chloramphenicol, methylparaben, and propylparaben in eye-drops. The limits of quantitation for all three analytes were below their respective working concentrations, proving the adequate sensitivity of the method. And the method was also robust for minor changes in the experimental conditions because the robustness study showed no significant variation in electrophoretic response and assay results of all the three analytes (Table 1).

In comparison to other published methods, the CZE method developed by Uysal et al. [11] gave better LOQs for methylparaben (about 1 *µ*g/ml) and propylparaben (about 0.85 *µ*g/ml) than those obtained in this work (5 *µ*g/ml) but using more complicated conditions (employing solid-phase extraction, internal standard, and UV detection at 200 nm) whereas another study using microchip electrophoresis with conductivity detection [12] gave lower LOQ for methylparaben (0.7 *µ*g/ml) but higher LOQ for propylparaben (6.0 *µ*g/ml) comparing to this work. Better sensitivity for chloramphenicol was also achieved with electrochemical detection (LOD about 0.3 *µ*g/ml) [8]. However, there was no available method for simultaneous assay of chloramphenicol, methylparaben, and propylparaben in eye-drops before this study, and this work was successful in providing a simple, easy-to-use method with sufficient reliability and sensitivity and without the need of elaborate sample preparation and being feasible on a commercial instrument for routine quality control. The suitability of this method was also proved by the results obtained on real eye-drops samples (Table 2). These results showed that these eye-drops meet common requirements of the content of active ingredient (95–105% of the labeled amount) and preservative (80–120% of the labeled amount) for pharmaceutical products.

## 4. Conclusion

In this study, a green, cost-effective, and simple CZE method has been developed for simultaneous assay of chloramphenicol, propylparaben, and methylparaben in eye-drops. The method was validated according to current requirements, and its reliability, through the studies of specificity, linearity, precision, accuracy, and robustness, was proved as acceptable for the intended application. Results of this study also confirmed the possibility and the need of promoting “green chemistry” in drug quality control activities, through the employment of analytical techniques friendly with the environment like capillary electrophoresis.

## Figures and Tables

**Table 1:** Summary of validation results.

Parameter	Chloramphenicol	Propylparaben	Methylparaben
*System suitability*			
** **Migration time (RSD (%), *n* = 6),	1.1	1.1	1.2
** **Peak area (RSD (%), *n* = 6)	1.3	1.5	1.5

*Linearity*			
** **Concentration range (*µ*g/ml)	1202.7–2806.2 7	6.7–15.7	55.5–129.5
** **Calibration curve	*y* = 2.3531*x* + 163.2	*y* = 7.3451*x* + 1.27	*y* = 8.747*x* + 58.58
** ** *R* ^2^	0.9984	0.9991	0.9990

Repeatability (RSD (%), *n* = 6, 1 day)	1.0	1.2	1.3
Intermediate precision (RSD (%), *n* = 12, 2 days)	1.6	1.3	1.4
Precision requirement, in RSD (%), of AOAC International [14]	≤3.7	≤7.3	≤5.3
*Accuracy (n* *=* *3 at each level)*			
** **At 80% of working concentration			
** **+Recovery range (%)	97.2–100.0	95.8–99.1	98.6–100.8
** **+RSD (%)	1.5	1.7	1.1

** **At 100% of working concentration			
** **+Recovery range (%)	98.7–100.1	100.6–104.3	94.8–96.7
** **+RSD (%)	0.7	1.8	1.0

** **At 120% of working concentration			
** **+Recovery range (%)	99.1–101.5	97.2–100.5	95.5–97.5
** **+RSD (%)	1.3	1.7	1.1

Accuracy requirements, in recovery rate (%), of AOAC International [14]	95–105	80–110	90–107
Limit of quantitation (µg/ml)	20.0	5.0	5.0

*Robustness*			
** **Variation of voltage (3 levels, *n* = 3 for each level)			
** **+RSD (%) of peak area	≤0.9	≤1.8	≤1.8
** **+RSD (%) of assay results	≤0.8	≤1.8	≤1.6

** **Variation of injection pressure (3 levels, *n* = 3 for each level)			
** **+RSD (%) of peak area	≤0.9	≤1.6	≤1.7
** **+RSD (%) of assay results	≤0.9	≤1.8	≤1.5

One-way ANOVA for assay results using different experimental conditions	*P* = 0.948 > 0.05	*P* = 0.945 > 0.05	*P* = 0.907 > 0.05

**Table 2:** Assay results of eye-drops sample.

Analytes	Chloramphenicol	Propylparaben	Methylparaben
Labeled amount	0.4% (32 mg/8 ml)	0.002% (0.16 mg/8 ml)	0.018% (1.44 mg/8 ml)
Average content (%, comparing to labeled amount, *n* = 6)	98.1	104.6	102.7
RSD of content (%)	1.0	1.2	1.3

## Data Availability

The data used to support the findings of this study are available from the corresponding author (ledinhchi@gmail.com) upon request.
